# Patient- and Caregiver-Informed Considerations for the Design and Implementation of Generative AI–Supported Patient-Centered Clinical Decision Support: Qualitative Study

**DOI:** 10.2196/75851

**Published:** 2026-07-14

**Authors:** Priyanka J Desai, Angela Dobes, Avantika S Shah, Jessica S Ancker, Lindsay Abdulhay, Sagarika Das, Caroline Peterson, CDSiC Trust and Patient-Centeredness Workgroup, Karim Hanna, Prashila Dullabh

**Affiliations:** 1 Health Sciences Department, NORC, University of Chicago, 1828 L Street NW, Washington, DC, 20036, United States, 1 301-634-9300; 2 Crohn's and Colitis Foundation, New York, NY, United States; 3 Department of Biomedical Informatics, Vanderbilt University, Nashville, TN, United States

**Keywords:** generative artificial intelligence, patient-centered clinical decision support, patient-centered care, patient engagement, qualitative study

## Abstract

**Background:**

Generative artificial intelligence (AI) has the potential to impact health care by transforming workflows and improving outcomes. Patient-centered clinical decision support (PC CDS) are digital tools that use patient-specific information and patient-centered outcomes research to improve health care decision-making. Generative AI is increasingly being incorporated into PC CDS tools. As patient-facing digital tools continue to expand within the health ecosystem, it is important to gather patient and caregiver perspectives about engaging with generative AI–supported PC CDS tools.

**Objective:**

This study aimed to generate a prioritized list of patient- and caregiver-informed considerations for the design, implementation, and use of generative AI in PC CDS.

**Methods:**

We conducted 6 small group discussions across 2 phases, with a total of 16 participants comprising patient and caregiver advocates. The first phase explored perspectives on generative AI–supported PC CDS. Using an iterative qualitative approach, we synthesized themes after each session to monitor saturation, which informed the development of an initial list of 7 key considerations for the implementation and use of generative AI–supported PC CDS. During the second phase, we generated a refined list of considerations using a prioritization ranking activity and peer validation approach.

**Results:**

Participants believed that generative AI–supported PC CDS tools have the potential to enhance efficiency, support clinicians, and improve health care decision-making but recognized that they could introduce challenges. Trust and willingness to use these tools are shaped by individuals’ health care experiences and familiarity with technology. Concerns included transparency, data security and accuracy, and potential for bias and mistrust in health care. Participants emphasized the importance of customizability and seamless integration into patient-clinician interactions. The tools’ success depends on clinicians’ skills and use. Our final list of 7 considerations includes the development of standards and design principles for generative AI–supported PC CDS tools, co-design with end users, considerations for mistrust in the health care system, monitoring and evaluation to ensure accuracy, education and training to understand and use AI, use of generative AI–supported tools that complement clinicians’ work and uphold the patient-clinician relationship, and AI that holistically uses patient data and tailors outputs.

**Conclusions:**

This study offers a patient- and caregiver-informed set of considerations for generative AI–supported PC CDS including holistic data use, continuous monitoring, addressing mistrust, and ensuring human oversight to mitigate risks such as errors in AI outputs. Participants emphasized the importance of co-design and the need for education, training, and user choice to support meaningful engagement. These interconnected considerations can inform future research and guide the design and implementation of patient-facing AI-supported PC CDS tools.

## Introduction

Artificial intelligence (AI) has rapidly evolved [1] for use in health care and medicine and has the potential to improve outcomes for patients and support clinicians in their work [2]. Generative AI, a subset of AI, comprises algorithms trained on large datasets of text, images, video, and audio that create new content, process information, and communicate using natural language [3]. Generative AI can shift activities from humans to computers for streamlined diagnosis processes, reduced costs and time, and improved outcomes for patients and clinicians [4,5]. Clinical decision support (CDS) encompasses digital tools that facilitate the delivery of timely information for health care decision-making among patients, clinicians, caregivers, and others [6]. CDS tools are found in electronic health records, patient portals, and mobile apps, and help reduce health care costs, improve efficiency, increase quality of care, and prevent errors or adverse events [6,7]. When combined with patient-centered considerations—family or caregiver support, community and culture, health concerns and medical history, and priorities, preferences, and values—CDS is patient-centered [8]. Patient-centered CDS (PC CDS) empowers individual patients and their caregivers and clinicians through a spectrum of digital decision-making tools that harness patient-centered outcomes research and patient-specific information [9]. Essentially, PC CDS encompasses a spectrum of decision-making tools that significantly incorporate patient-centered factors related to knowledge, data, delivery, and use [10]. Knowledge refers to the use of patient-centered outcomes research findings or evidence-based findings from other types of research. Data focuses on the incorporation of patient-generated health data, patient preferences, social determinants of health, and other patient-specific information. Delivery refers to directly engaging patients and/or caregivers across different settings. Finally, use focuses on facilitating bidirectional information exchange in support of patient-centered care, including shared decision-making [11].

Leveraging generative AI for PC CDS can improve health care decision-making and its related procedures and processes [11-15]. A few examples of AI-supported PC CDS include a tool [16] that supports physician workflow and diagnostic imaging precision, an AI-supported application designed to answer commonly asked medical questions [17], a chatbot-based platform that supports insurance plan members [18], and automation tools that streamline routine tasks such as scheduling [19] and generating discharge summaries [20].

As generative AI is becoming increasingly used in health care, the safety, trustworthiness, and transparency of these generative AI–supported tools are ever more important. To ensure patient trust, it is important that the use of generative AI in PC CDS considers the perspectives of patients and caregivers. While the literature on AI in health care is rapidly expanding, we noted 2 key gaps in the literature. First, previous studies about patient or public attitudes often broadly probe general use in health care, use for diagnoses, or clinician use [21-24]. Given the increasing importance of patient-facing digital tools within the health technology ecosystem, it is important to gather patient perspectives about directly engaging with AI-supported tools for health care decision-making. Second, while previous consensus-based efforts have resulted in recommendations for the use of AI in CDS [25], we lack patient-driven recommendations that acknowledge patients as potential CDS end users. Notably, previous reviews have found that patient perspectives remain insufficiently incorporated into AI design and deployment guidance and call for additional qualitative research on patients and caregivers [26,27].

This paper addresses these gaps and extends previous research by focusing specifically on patient and caregiver perspectives on the use of generative AI in PC CDS [28]. By gathering patient and caregiver perspectives, we aimed to generate a prioritized list of patient- and caregiver-informed considerations for the design, implementation, and use of generative AI in PC CDS.

## Methods

### Study Design and Participant Selection

We conducted 6 qualitative small group discussions held in 2 phases with 16 participants (7 participants in phase 1 and 9 participants in phase 2) to elicit patient and caregiver representatives’ perspectives on the use of generative AI–supported PC CDS, factors impacting trust, implications for patient-clinician relationships, and considerations for design and implementation of these tools. Ten participants identified as patient advocates, 5 as caregiver advocates, and 1 as both. We included patient and caregiver partners to reflect their shared and complementary roles in health care decision-making.

We used purposive, convenience sampling to identify patient or caregiver advocates with lived experiences. Specifically, we first leveraged existing relationships with established patient and caregiver networks to identify individuals with lived experience. We then expanded the sampling frame by systematically searching publicly available patient and caregiver advocacy resources (eg, National Association of Healthcare Advocacy directory) to identify additional prospective participants. Peer referrals were used to further broaden the pool. Participant outreach for recruitment was conducted virtually using standardized email invitations and follow-up messages. A virtual format was used to support the flexible scheduling and participant convenience.

### Discussion Guide Development

All discussions used a semistructured discussion guide to gather participants’ perspectives on generative AI use in PC CDS. For the first phase of data collection (phase 1), the guide featured 2 generative AI application use case scenarios in PC CDS (Textbox 1), adapted from pilot PC CDS tools that were under development at that time [29,30]. Given the varying public understanding of AI and how it can be used [31], we developed this scenario approach to ensure that participants understood health care AI and to focus their discussions. We conducted three 60-minute virtual cognitive debriefings [32] with 1 patient representative and 2 researchers to refine the guide for clarity and comprehension. During these sessions, respondents answered each discussion question and provided feedback on question phrasing, commonly used terms (eg, artificial intelligence and clinical decision support) and illustrative examples, and language in the use case scenarios. They also reviewed a background document on PC CDS and AI intended for use with discussion participants. The discussion guide for phase 1 began with soliciting general perspectives on the use of AI in health care. We then discussed the potential benefits and concerns related to the use of the 2 generative AI application use case scenarios in PC CDS. The guide included probing questions to invite participants to share their level of trust in these tools along with influencing factors and the impact of the tools on the patient-clinician relationship.

Textbox 1. **Illustrative examples for generative artificial intelligence–supported patient-centered clinical decision support shared with small group discussion participants.**

**Example 1:** Patient follow-up question application.

- A patient asks a question about their cough in their online patient portal. An artificial intelligence (AI)–supported tool uses evidence-based information to generate follow-up questions in real time and collect additional information about the patient’s concern. Once the tool has gathered enough information from the patient, it summarizes the information and shares the summary with the doctor. The doctor then moves forward with any next steps needed to address the cough, such as ordering a laboratory test, sending a prescription to the pharmacy, or asking them to come into the office.

**Example 2:** AI-supported intervention to support medication adherence.

- To help patients remember to take their newly prescribed medications consistently, an AI-supported application automatically sends check-in messages to patients. The application communicates with the patient via text messages using the patient’s selected language, such as English or Spanish. The application sends medication reminders and generates questions about any new symptoms. By using the patient’s responses and asking relevant follow-up questions, the application can then provide updates to the patient’s doctors, nurses, or other care team members so that they can monitor the patient and make any recommendations or changes.

### Data Collection

#### Overview

Data collection occurred in 2 phases: January and February 2024 and November 2024. All small group discussions were virtual and lasted 60‐75 minutes. Prior to each discussion, participants received a 2‐ to 3-page background document on generative AI and PC CDS. Participants provided verbal consent for participation in and recording of virtual interviews, with the option to skip questions or exit at any time. Participants were offered a gift card incentive for their participation in study activities.

#### Phase 1

We conducted 3 small group discussions across 7 participants (5 patient advocates and 2 caregiver advocates) to understand their perspectives on the use of AI in health care and the impacts of AI on patient and caregiver trust in providers and health care systems. From phase 1 discussion results, we generated a list of 7 considerations for the implementation of generative AI for PC CDS [33]. Textbox 2 provides the initial list of considerations for the implementation and use of AI in PC CDS.

Textbox 2. **Initial considerations for the implementation and use of artificial intelligence in patient-centered clinical decision support.**

- Consult patients and provide choices when introducing artificial intelligence (AI) tools for patient-centered clinical decision support (PC CDS).
- Provide education and training to support patients’ and caregivers’ use and understanding of AI-enabled tools.
- Develop standards and design principles to promote transparent, safe, and secure implementation of AI in health care.
- Use AI as a supplementary tool to support and strengthen clinicians’ work.
- Conduct continuous monitoring, critical appraisals, and due diligence when implementing generative AI technologies for PC CDS.
- Consider how to holistically use all data to personalize and tailor AI outputs in the development of AI-enabled PC CDS.
- Consider challenges related to mistrust and navigating the existing health care system, particularly for historically marginalized or vulnerable populations.

#### Phase 2

We used a peer validation approach [34,35], where we presented the list of 7 considerations generated in phase 1 to a new set of 9 participants (5 patient advocates, 3 caregiver advocates, and 1 who identified as both a patient and a caregiver advocate) across 3 small group discussions. These participants were selected from the same sampling frame as those in the phase 1 discussions to further refine the considerations identified during the first phase discussions and identify new considerations. Before the discussions, participants completed a prioritization activity in Qualtrics to rate and rank the 7 considerations identified during phase 1 [32]. The activity used a 5-point Likert scale of importance ratings to rate each consideration individually (ie, 1=not at all important and 5=extremely important). Participants also ranked all 7 considerations from 1 to 7 to indicate relative importance (1=most important and 7=least important).

We then adapted the original semistructured discussion guide, sharing the illustrative generative AI PC CDS examples (Textbox 1) to explore the prioritization rankings and to identify any additional considerations participants felt should be added to the list. Participants were provided with a copy of their rankings before the discussions. During the discussion, the facilitator walked participants through the results. This enabled participants to reflect on the group’s collective views and provided an opportunity for further discussion and refinement of the results. Following the small group discussions, participants who completed the prioritization activity were asked to complete a final ranking exercise to indicate the relative importance of a refined list of 7 considerations.

### Analysis and Synthesis

We conducted a rapid qualitative content analysis of the transcript-style notes to inductively identify key themes [36]. This approach allowed us to explore participants’ perspectives in a flexible and open-ended way, without predefined categories, ensuring that the analysis reflected their lived experiences and views. We used the discussion guide structure to inform deductive coding to identify initial themes and patterns and then inductively coded the findings to identify additional themes. As part of our rapid qualitative approach, the research team synthesized themes immediately after each small-group discussion, allowing us to monitor thematic development in real time and assess when code saturation had been reached (ie, no new themes or concepts were identified within the sampled population and themes remained stable across discussion groups), consistent with established qualitative research guidance on iterative analysis and saturation [37].

The themes from the phase 1 discussions were synthesized by 5 team members to form the initial considerations. These findings were iteratively refined based on review from the broader team. Similar to the initial phase, 5 team members identified additional themes from phase 2, which informed revised considerations for the design and implementation of generative AI–supported PC CDS. Across both phases, we continued patient and caregiver recruitment until thematic saturation was reached within the sampled population, indicating that additional interviews were unlikely to yield new insights [38,39].

Finally, a series of collaborative discussions was conducted to refine the new themes and finalize the list of considerations before the final ranking activity. In parallel with the qualitative analysis, we used descriptive statistics to determine the average and range of ranks for each list (initial and final) of considerations shared with participants during phase 2.

### Ethical Considerations

This study underwent review by the National Opinion Research Center at The University of Chicago Institutional Review Board (FWA00000142). The project was determined to be non–human subjects research. Participants provided verbal consent before participating in and being recorded during virtual interviews. Participants were informed that they could decline to answer any question or withdraw from the interview at any time. As a token of appreciation, participants were offered a US $50 gift card, which was distributed via email following the interview. To protect participant confidentiality, all interview transcripts were deidentified prior to analysis, and no identifying information was included in the analytic dataset. We adhered to the SRQR (Standards for Reporting Qualitative Research) checklist when drafting this manuscript [40].

## Results

### Overview

Participants represented a range of demographic backgrounds and conditions and reported varying familiarity with AI-supported digital health tools. The sample included individuals who are Black (3 participants), Native Hawaiian or Other Pacific Islander (1 participant), and 11 women. Ages of participants ranged from late 30s to mid-70s. Five participants were cancer survivors. Other conditions for patient advocates included lupus, multiple sclerosis, and other autoimmune diseases, as well as rare diseases. Caregivers cared for family members with dementia and cancer.

In the following section, we first describe key themes related to general perspectives on generative AI–supported PC CDS tools identified during phase 1 with 7 participants. Next, we present findings from the prioritization activity and additional themes that emerged during phase 2 with 9 participants. Finally, we describe the refined list of considerations.

### Key Themes From Phase 1 Small Group Discussions

Perspectives on the use of generative AI in health care and for PC CDS ranged from skeptical to optimistic. In responding to the scenarios, participants provided a range of views, including advantages, disadvantages, and recommendations for the use of AI in PC CDS.

*Generative AI–supported PC CDS could be beneficial for improving accuracy and efficiency in health care*: Participants believed that generative AI–supported PC CDS could potentially strengthen clinicians’ practice and improve patient-clinician communication, which may reduce unnecessary health care visits and optimize time during in-person appointments. One participant shared:

*Coming from my own personal experience...something like this would be super helpful for me in that process in which I don't have to wait 3 to 6 months to see my doctor for them to talk to me...So, I feel like in that way that this would be a really useful tool for me and a lot of other people with chronic conditions*.

[Discussion 2]

Participants had no concerns with using generative AI–supported tools to support health care decision-making if human oversight remained and clinician judgment was not replaced.

*Generative AI–supported PC CDS could also introduce new inefficiencies, communication challenges, and additional barriers to personalized health care delivery*: Participants noted that patients may be asked to reshare information collected by a generative AI–supported PC CDS tool if their clinician does not review it—counteracting AI’s time-saving potential. Additionally, the use of AI in health care may introduce communication barriers, and participants noted that patients should be provided options to opt out of using the tool or connect with a human. One participant noted:

*It’s like a human relationship. There comes a time in some interpersonal relationships where one party needs to say, “This isn't working for me.” You need to be able to say that to the chatbot*.

[Discussion 1]

While such tools can address basic health issues, some expressed concern around generative AI–supported PC CDS tools’ capacity to respond to more serious, complex health problems. One participant said:

*To get a proper diagnosis, it takes looking at [a] person’s vast medical history and there just isn't enough time at the doctor’s office for a doctor to do that once. You know, you've got years and years and years of [medical history] information. I don't know that the AI is going to be able to access any of that information*.

[Discussion 2]

They raised potential limitations about AI’s ability to successfully integrate a patient’s entire health history and offer personalized recommendations through generative AI–supported PC CDS.

*Willingness to trust and use generative AI–driven PC CDS may be influenced by factors including individual health care experiences and familiarity with health care technology*: Some participants shared previous challenges interacting with the health care system, including years of misdiagnosis despite having access to care teams. Experiences such as receiving inaccurate answers to a simple medical question when using a general AI tool led to skepticism about the potential of generative AI–supported PC CDS to combat underlying issues in the health care system or patient-facing health care technology (eg, malfunctioning patient portals).

Several participants stressed the need for clear information on its purpose and benefits for patients and caregivers while the tool is being introduced. They highlighted that patients and caregivers should receive education and training on generative AI tools, especially patients and caregivers with low health and digital literacy or limited experience using health technology to ensure adoption.

Participants noted that trust and use of generative AI–supported PC CDS depend on the accuracy of the data informing the tool. For example, a few participants noted that if generative AI–supported PC CDS relies on outdated or non–evidence-based sources, it could spread misinformation and result in their mistrust of the device. Most participants also warned that biased data could worsen health care disparities and impact trust, particularly for vulnerable populations.

*Health care organizations must prioritize transparency with generative AI–supported PC CDS*: For instance, participants highlighted the importance of clear user interface design to distinguish AI from human interaction. Several participants described concerns about the security of data that are shared and stored in AI tools. A participant noted:

*I have concerns about the way it’s built. I have concerns about what it’s consuming, how it’s learning. I have concerns about where the data is going and what it’s doing with it*.

[Discussion 3]

For the purposes of transparency and confidence regarding the accuracy of generative AI–supported PC CDS tools, participants expressed the need for disclaimers clearly stating who owns the tool and has access to the tool’s information, information on underlying data sources, and how collected data are stored and used.

*Clinicians’ use of generative AI–supported PC CDS to support decision-making does not impact patients’ view of them*: Instead, how the tools are used by their clinician was viewed as more influential in shaping the patient-clinician relationship and the quality of care they receive. For example, a participant explained:

*I think AI is a great collaborative tool for clinicians. I am one of those people who does not want...to see it go beyond being a collaborative tool*.

[Discussion 2]

Generative AI–supported PC CDS tools should benefit patients, caregivers, and clinicians, and participants emphasized that clinicians should not be the only users who are positively impacted by their use. To support patient-centeredness, patients and caregivers should have input on the design and implementation of generative AI–supported PC CDS tools.

*Health care organizations should avoid generative AI–supported PC CDS that disrupts patient-clinician interactions*: For instance, some participants stressed the importance of using generative AI–supported PC CDS to supplement, not replace, patient-clinician interactions, emphasizing that clinicians should still follow up directly with patients about their health. Participants also noted that their perception of their clinician would be negatively impacted if they were to overly rely on AI outputs. A participant shared:

*I don’t want this to be something that is completely left up to artificial intelligence...AI is only as good as the data that it is drawing from...Clinicians are only as good as what they learn from [previous] experiences, and the clinicians themselves continue to grow. I think AI can be a part of that, but certainly not a replacement*.

[Discussion 2]

As a result, most participants emphasized that the safe, effective use of generative AI–supported PC CDS requires human oversight and ongoing confirmation that it is improving health care. As noted in the “Methods” section, the themes described in this section resulted in the initial list of considerations shown in Textbox 2.

### Phase 2 Prioritization of Initial Considerations and Peer Validation

Before small group discussions, the 9 phase 2 participants rated the importance of the initial list of considerations on a Likert scale.

#### Initial Importance Ratings

**Figure 1.**
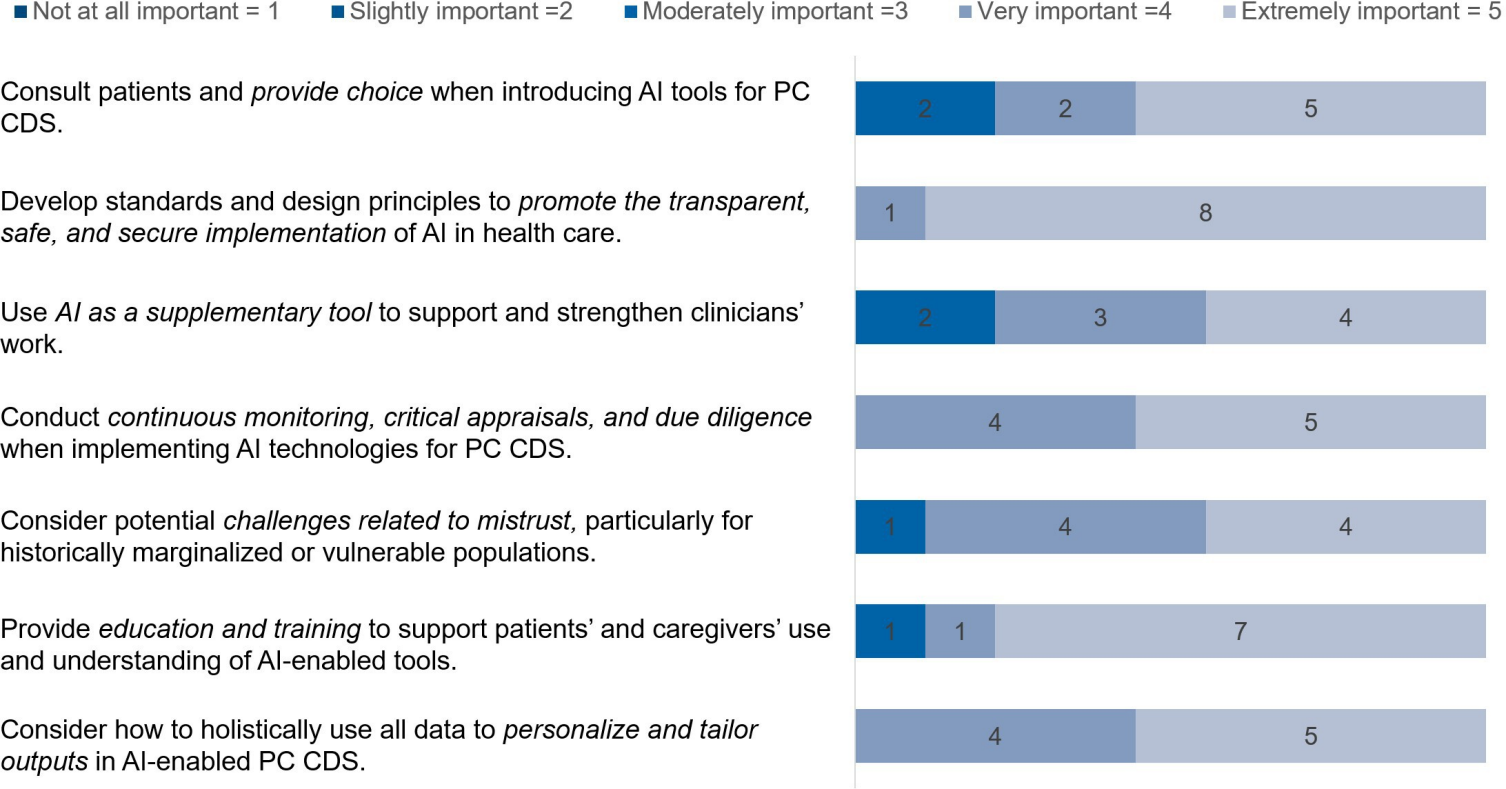
Importance rating (on a 5-point Likert scale) for the initial list of 7 considerations (N=9). The numbers in the bars represent the number of participants who selected the Likert scale response for consideration. AI: artificial intelligence; PC CDS: patient-centered clinical decision support.

Figure 1 shows the initial list of considerations and their importance ratings. The consideration “Develop standards and design principles to promote the transparent, safe, and secure implementation of AI in healthcare” was the only consideration that 8 of 9 participants rated as extremely important (no consideration was rated as extremely important by all participants). None of the considerations were rated as “Not at all important” or “Slightly important.”

#### Initial Ranking

Table 1 shows average rank and range of rank scores selected by participants for each consideration. There was significant variation in ranking for each consideration. Several participants viewed each consideration as fundamental to the successful implementation of generative AI–supported PC CDS and saw them as interrelated. For instance, implementing appropriate standards and design principles could lead to continuous monitoring, which would help mitigate bias and address issues of mistrust. Furthermore, they highlighted that lower-ranked considerations might be addressed if higher-ranked considerations are implemented effectively.

**Table 1.** Average rank and range for initial list of considerations (rank 1=most important and rank 7=least important).

Initial list of considerations	Average rank	Range
Consult patients and provide choice when introducing AI^a^ tools for PC CDS^b^.	2	1-6
Develop standards and design principles to promote the transparent, safe, and secure implementation of AI in health care.	3	1-6
Use AI as a supplementary tool to support and strengthen clinicians’ work.	4	1-7
Provide education and training to support patients’ and caregivers’ use and understanding of AI-enabled tools.	4	2-7
Consider potential challenges related to mistrust, particularly for historically marginalized or vulnerable populations.	4	2-7
Conduct continuous monitoring, critical appraisals, and due diligence when implementing AI technologies for PC CDS.	5	2-7
Consider how to holistically use all data to personalize and tailor outputs in AI-enabled PC CDS.	5	2-7

a AI: artificial intelligence.

b PC CDS: patient-centered clinical decision support.

### Key Themes Regarding Generative AI in PC CDS From Phase 2

In the phase 2 small group discussions, participants echoed many of the themes identified in phase 1 (Textbox 3):

Textbox 3. **Themes identified from phase 2.**

  - Generative artificial intelligence (AI)–supported patient-centered clinical decision support (PC CDS) has a promising role in improving health care efficiency and outcomes by summarizing health histories, streamlining communication, managing medications, providing information, supporting shared decision-making, and triaging patient and caregiver needs.
  - Implementation of generative AI in PC CDS should be guided by principles of transparency and security with particular attention to data use, data provenance, and cybersecurity.
  - Health care systems must consider the needs of vulnerable populations who may have more trust issues with health care.
  - Patients must always be aware that they are interacting with AI and not a human.
  - The provision of education and training on generative AI–supported tools for patients and caregivers is fundamental to building trust and ensuring the appropriate usage.

Phase 2 participants also raised 5 additional themes related to the use of AI in PC CDS outlined in the following section.

*The potential for poor quality data and other flaws requires that generative AI–supported PC CDS be regularly reviewed for accuracy*: Participants raised concerns about the risks associated with the use of generative AI. Insufficient and poor-quality data input could lead to errors in AI-generated outputs, undermining trust in these tools. Ensuring that the data informing generative AI–supported PC CDS are accurate and reliable was emphasized as essential to mitigating these risks. Participants stressed that AI-generated outputs should be reviewed by clinicians and medical experts to verify information before the outputs are shared with patients (eg, confirming an AI-generated symptom summary accurately reflects what the patient is experiencing). Accuracy is essential for fostering confidence in AI-supported PC CDS and ensuring its adoption.

*All evidence-based, science-based information has to be paramount...because you don’t want to get into a situation where someone is just creating information that’s just not accurate. There has to be [underlying] comfort [when using the tool] and accurate information coming for the patient*.

[Discussion 4]

*Generative AI–supported PC CDS must be designed for usability by incorporating human-centered design methods and principles*: Participants emphasized that developing generative AI–supported PC CDS tools with human-centered design principles is essential for ensuring usability, particularly for individuals with low health and digital literacy. They advocated for training and education to support both patients and clinicians in adapting to AI technologies, fostering greater accessibility and trust.

*I think human centered design is essential, not just on the patient and caregiver side, but on the clinician care team side. It’s very easy to think things are going to work a certain way, and I know, with some other clinical decision support tools sometimes when the answer comes back for a clinician, if it doesn’t jive with their heuristic or what they thought, they don’t quite know what to do with it*.

[Discussion 6]

*Generative AI–supported PC CDS should be customizable to accommodate varying needs and preferences*: Participants noted that generative AI–supported PC CDS that relies on a user’s ability to read and write may deter certain patients and caregivers from uptake. Participants emphasized that generative AI–supported PC CDS should provide options for users to select their preferred method for providing and receiving information to enhance comfort and trust. Suggested features included allowing users to choose a chatbot’s voice, accent, or language. A participant explained:

*I would like to see at least a few languages available. But let’s say if it’s just in English, is [there] the ability to give that bot the option to read it to you rather than to write it to you?...when you think of the elderly or people with dementia or people who are older and caring for someone or stressed out during an emergency situation, having it read to you and having it repeat your answer is a way to confirm that this is actually what you’re asking*.

[Discussion 4]

*Generative AI–supported PC CDS could support caregivers to better manage care as health care surrogates*: Participants shared that generative AI–supported PC CDS tools could better facilitate caregiver participation and involvement in shared decision-making, especially for those with cognitive impairments. For instance, caregivers might prefer to use generative AI–supported PC CDS tools to address simpler health care situations experienced by their loved ones, such as medication reminders or asking about a runny nose.

*I think you gave a great example on how it can help caregivers with, medication management, doing those reminders and some of those routine tasks...can be helped by having...the chat bot automatically do it*.

[Discussion 4]

*The effective use of generative AI–supported PC CDS may be tied to the skills of the clinicians using them*: Participants noted that the promise of AI alone is not enough to overcome deficiencies in human factors that are crucial for health care delivery such as clinician knowledge and communication skills. As one participant shared:

*With doctors who are not good at communication, [it] goes both ways: they’re not good at communicating to the patient; they’re not good at listening to the patient either...So, I’m not sure that the AI in this case is going to make the difference*.

[Discussion 5]

Although generative AI–supported PC CDS has the potential to complement and strengthen clinicians’ practice, its overall impact is highly dependent on the clinician’s communication and engagement skills. This sentiment was echoed by participants who expressed skepticism about the ability of such tools to substantially alter clinicians’ approach and delivery of care.

### Refined List of Considerations for Design and Implementation of Generative AI–Supported PC CDS Tools

Phase 2 small group discussions provided insights that informed updates to the initial considerations (including the addition of a new consideration), ensuring greater alignment with patient and caregiver perspectives. Furthermore, the actions encompassed within each consideration were clarified, reflecting the discussions about factors influencing trust and patient-centeredness. Table 2 shows the revised implementation considerations for generative AI–supported PC CDS along with final rankings.

**Table 2.** Average rank and range for final patient- and caregiver-informed considerations for design and implementation of generative artificial intelligence–supported patient-centered clinical decision support (rank 1=most important and rank 7=least important).

Final list of considerations	Average rank	Range
Develop standards and design principles to promote the transparent, safe, and secure implementation of generative AI^a^ in PC CDS^b^.	2	1-5
Co-design and test generative AI–supported PC CDS with patients and clinicians, and consult patients before implementation of such tools.	2	1-5
Provide education, training, and choice to support patients’ and caregivers’ understanding of their benefits and use of generative AI–supported PC CDS tools.	4	2-7
Consider potential challenges related to mistrust and navigating the existing health care system, particularly for underserved populations.	5	2-7
Use generative AI–supported PC CDS as a complementary tool to support clinicians’ work and enhance patient communications and relationships.	5	3-6
Consider how to holistically use all data to personalize and tailor outputs in generative AI–supported PC CDS.	5	2-7
Conduct continuous monitoring, critical appraisals, and due diligence when implementing generative AI technologies for PC CDS.	5	3-7

a AI: artificial intelligence.

b PC CDS: patient-centered clinical decision support.

*Develop standards and design principles to promote the transparent and secure implementation of generative AI in PC CDS*: Standards to develop and implement generative AI–supported PC CDS tools are fundamental. Moreover, health care organizations encouraging patients to use these tools should design them to provide clear information, that is, use lay terms, regarding the tools’ data use and ownership, cybersecurity, and privacy, to help patients feel safe and address their potential concerns. This includes transparency about the underlying data used to inform and train generative AI–supported tools. Patients and caregivers should also have the opportunity to review information and terms prior to use and have ongoing, easy access to this information should they wish to reconsider. When interacting with generative AI–supported PC CDS tools, participants emphasized that they would want to be able to clearly recognize when an AI is communicating with them rather than a human.

*Co-design and test generative AI–supported PC CDS with patients, caregivers, and clinicians, and consult patients before implementation of such tools*: Patients, caregivers, and clinicians should be involved in collaboratively designing and testing generative AI–supported PC CDS before implementation to ensure that tools are built with the needs of patients and clinicians in consideration. This will ensure that the design and usability cater to a range of preferences, including how information is displayed (text, visual, and language) and the functionality of the tool. Before implementing generative AI–supported PC CDS tools, health care delivery systems should seek feedback and input from patients and caregivers, potentially through advisory groups or community settings.

*Provide education, training, and choice to support patients’ and caregivers’ understanding of their benefits and use of generative AI–supported PC CDS tools*: It is important to provide patients and caregivers with thorough training and education on the effective use of generative AI–supported tools. Given the prevalence of misinformation online, participants noted that generative AI–supported PC CDS can pose risks for individuals unfamiliar with its proper usage. Moreover, not all patients will know how to ask the right questions when interacting with generative AI–supported PC CDS. Education and training on generative AI–supported PC CDS will support the proper and effective use of this technology by patients and caregivers.

It is important to provide patients and caregivers with thorough training and education on the effective use of generative AI–supported tools. Given the prevalence of misinformation online, participants noted that generative AI–supported PC CDS can pose risks for individuals unfamiliar with its proper usage. Moreover, not all patients will know how to ask the right questions when interacting with generative AI–supported PC CDS. Education and training on generative AI–supported PC CDS will support the proper and effective use of this technology by patients and caregivers.

It is also crucial to give patients the option to opt out of using generative AI–supported PC CDS, particularly after they have had a chance to understand and try them. Some participants suggested implementing a “help desk” or simple mechanism to bypass the AI and connect directly with a care team member.

*Consider potential challenges related to mistrust and navigating the existing health care system, particularly for underserved populations*: Health systems need to consider their patients’ socioeconomic backgrounds when implementing generative AI–supported PC CDS. Factors such as limited health literacy, access to technology, and overall comfort with AI are potential barriers to enabling its use by certain patient populations. Participants suggested that mistrust and cultural concerns with the health care system could significantly impact the ethical use of generative AI–supported PC CDS. Without considerations for historically marginalized or vulnerable populations, the implementation and use of these tools may contribute to mistrust in the health care system.

*Use generative AI–supported PC CDS as a complementary tool to support clinicians’ work and enhance patient communications and relationships*: Generative AI–supported PC CDS should not replace clinician oversight and judgment. Clinicians should use these tools to improve their practice by allocating more time to actively listen to patients or their caregivers, engaging in meaningful dialogues, and collaboratively using these tools to enhance clinical decision-making. Participants cautioned against overreliance on these tools, emphasizing that clinician involvement and oversight are essential.

*Consider how to holistically use data to personalize and tailor outputs in generative AI–supported PC CDS*: AI may have limited ability to access data across multiple electronic health record systems and tailor outputs to meet the unique needs of individual patients. Participants emphasized that each person is unique, with some having extensive health histories that are difficult to convey in a typical clinical setting. As a result, patients may face challenges with communicating detailed medical information to generative AI–supported tools. It is essential to implement concrete measures during the development of generative AI–supported PC CDS tools to facilitate the synthesis of entire patient health histories, ensuring that outputs are comprehensive and personalized for each patient.

*Conduct continuous monitoring, critical appraisals, and due diligence when implementing generative AI technologies for PC CDS*: To sustain and improve trust in such tools over time, health care delivery systems need to conduct critical, continuous evaluation of the tool’s functionality and ability to provide accurate, valuable, and reliable recommendations or findings. Several participants suggested that it is crucial to incorporate appropriate oversight procedures and safeguards to mitigate potential risks arising from the current known limitations of generative AI technology, such as errors in AI outputs. As the use of AI continues to grow in health care, ongoing monitoring and evaluation will help to support the quality and usefulness of generative AI–supported PC CDS tools. As part of the evaluation, input should be solicited from all users through feedback forms. Testing and evaluation need to begin early, prior to and during implementation, to ensure feedback.

## Discussion

Overall, participants valued generative AI–supported PC CDS tools’ potential to improve decision-making processes, reduce administrative burden, and streamline patient-clinician communication but raised concerns about safety, transparency, trust, underlying data sources, and preservation of human interactions. In the following section, we discuss the implications of our findings and contextualize them with prior work.

### Principal Results

We identified 7 key considerations related to the design and implementation of generative AI–supported PC CDS tools that are important to patients and caregivers. The considerations highlight the need to center patient, caregiver, and clinician perspectives in the development, implementation, and ongoing monitoring and improvements of generative AI–supported PC CDS tools. Transparency, safety, and security were paramount to ensuring trust of PC CDS tools, with attention to underserved patient populations and those with barriers to accessing and using technology. Participants emphasized that generative AI–supported PC CDS tools should complement, not replace, direct clinician interactions. Participants also suggested a range of design features and data accuracy to support trustworthiness and user adoption across a range of populations.

### Comparison With Prior Work

Our findings align with existing research on attitudes toward AI in health care, demonstrating both enthusiasm and reservations [9,12,23,24,30,41]. A nationally representative study found that most patients have positive views on AI in health [24]. However, this same study also found that most respondents raised concerns around the potential for misdiagnosis, privacy issues, reduced time with clinicians, and challenges among underserved groups. Like previous studies [42], participants in our study viewed generative AI–supported PC CDS as promising for health care decision-making and yet had concerns about accuracy, privacy, and preserving patient-clinician interaction. For example, a recent study on an AI-supported CDS tool for skin cancer screening found patients generally receptive to AI, provided it upheld the patient-clinician relationship [43]. Participants in our study emphasized transparency as crucial for building trust in generative AI–supported PC CDS, highlighting a need for clear information about the tool’s ownership, methodology, data security, and continuous monitoring. This aligns with related work on fostering patient trust when using AI in health care [44], and previous research [45] on credibility factors for PC CDS. Considerations for AI-based PC CDS tools identified in this study concur with a November 2024 paper that synthesized key recommendations for AI-supported CDS tools through a multistakeholder workgroup, including the need for model training, monitoring, and continuous evaluation to address challenges such as privacy and fairness [25]. However, while there is overlap between these recommendations and our considerations, an important differentiator is that participants of the previous effort identified “users” of AI-supported CDS as primarily clinicians. Our manuscript extends previous work by acknowledging that patients and caregivers are potential end users of AI-supported CDS.

Previous research has highlighted the need for additional information about patient and caregiver perspectives and feedback for the development and use of AI-supported digital health tools [27,46]. In using qualitative methods to ground findings in the lived experience, our study offers 3 important contributions. First, this paper moves beyond general attitudes to a prioritized set of patient- and caregiver-informed considerations tailored to generative AI–supported PC CDS workflows. To address the gap of high-level findings noted in prior reviews, we include quotations that surface trade-offs and conditions for trust (eg, transparency, opt-out, and oversight), enabling readers to understand context in participants’ own words. Second, our findings elevate co-design as critical for AI-supported patient-facing tools. While previous literature notes inconsistent co-design practice and reporting [47,48], our findings emphasize co-design and user testing with patients or caregivers as a core implementation requirement for patient-facing AI-enabled tools. Finally, our findings highlight the patient-clinician relationship as a foundation for acceptance and appropriate use of AI tools. Building on prior evidence [45,49] that acceptance depends on preserving the clinician relationship, our findings highlight that clinician-led introduction of generative AI–supported PC CDS is essential for building trust, and the tool’s positive impact depends on the clinician using it to collaboratively enhance patient communication and shared decision-making. Notably, the mere use of the tool neither significantly strengthens nor undermines the patient-clinician relationship. These findings have been less emphasized in prior work [45,50].

### Limitations

We used a convenience sample and therefore recognize that participants may not fully represent populations with limited health or digital literacy or historically underserved populations. Moreover, as patient and caregiver advocates, participants may possess a higher baseline digital and health literacy than the general patient population. Collection of additional demographic data and measurement of digital health literacy would support greater transferability of our findings.

Another limitation of this work is that the specific illustrative examples of AI-supported PC CDS tools (shown in Textbox 1) provided to initiate discussions may have shaped the areas of focus and influenced the perspectives expressed by participants. Different scenarios or discussion prompts (including other uses of generative AI and high-acuity situations) might have elicited alternative themes or concerns regarding the use of AI in health care, particularly for PC CDS. Finally, the data collection period was spread over 10‐11 months, and in that time, there was significant discussion in the public domain about the use of generative AI tools, which may have impacted participant perceptions and prioritizations in phase 2.

### Future Directions

Future efforts should prioritize integrating the identified patient- and caregiver-informed considerations when developing and implementing generative AI–supported PC CDS by identifying specific action steps for each of them. Such efforts will allow further examination of the interdependencies among the key considerations identified and the creation of integrated approaches that enhance both patient-centeredness and trust. As we move forward with the design and implementation of such tools, it is also important to gather perspectives from clinicians and health system leaders who have real-world experience with generative AI–supported PC CDS, as well as those involved in their co-design. Leveraging implementation science frameworks [51] can also support these efforts by identifying effective strategies for promoting the acceptability, scalability, and sustainability [52] of specific AI-supported PC CDS. Furthermore, it is critical to explore the ethical implications of PC CDS developed for a clinical scenario, focusing on issues such as algorithmic bias, access barriers, and the unique needs of the patient population served. Future studies should prioritize engaging participants with limited digital or health literacy or historically underserved populations to identify specific concerns, needs, and preferences regarding generative AI–supported PC CDS tools.

While this effort was not designed to differentiate between patients and caregivers, future research should also explore the differing needs and perspectives of patients and caregivers related to these tools. Collecting data from a range of patients and caregivers could offer insights across demographic factors and health conditions, allowing for meaningful comparisons between subpopulations. Finally, views on generative AI–supported technologies are likely to evolve over time, given the relative novelty of these tools. As public awareness, experiences, and attitudes toward AI in health care grow, perspectives on AI-supported PC CDS will continue to change. This underscores the importance of ongoing research to capture shifts in patient and caregiver attitudes as AI becomes more integrated into health care systems.

### Conclusions

This paper contributes to the growing literature on patient and caregiver perspectives as potential end users of generative AI–supported PC CDS. The considerations identified emerged through a 2-phased approach in which themes generated in phase 1 shaped the structure of phase 2 discussions and prioritization exercises, enabling participants to revisit, refine, and rank the considerations that emerged earlier. The final considerations included using data holistically to personalize outputs, continuously monitoring and appraising AI tools, and addressing challenges such as mistrust. Additional considerations highlighted the importance of co-designing and testing AI tools with both patients and clinicians to ensure that these tools complement, rather than replace, clinician decision-making. Finally, participants also stressed that providing education, training, and choice will help patients and caregivers better understand and engage with AI tools. Participants described the considerations as interconnected, emphasizing that each consideration is essential to the successful design and implementation of AI-supported PC CDS.

## Abbreviations:

AI: artificial intelligence
CDS: clinical decision support
HHS: United States Department of Health and Human Services
PC CDS: Patient-Centered Clinical Decision Support
SRQR: Standards for Reporting Qualitative Research
